# Aplicación del Modelo de Adaptación de Roy en el contexto comunitario* **

**DOI:** 10.15649/cuidarte.3016

**Published:** 2023-12-20

**Authors:** María Alvarado García, Blanca Cecilia Venegas Bustos, Alejandra Ángela María Salazar Maya

**Affiliations:** 1 . Universidad Antonio Nariño, Bogotá. Colombia Email: alalvarado39@uan.edu.co Universidad Antonio Nariño Universidad Antonio Nariño Bogotá Colombia alalvarado39@uan.edu.co; 2 . Universidad de la Sabana, Chía Bogotá Colombia. E-mail: blancac.venegas@unisabana.edu.co Universidad de la Sabana Universidad de la Sabana Bogotá Colombia blancac.venegas@unisabana.edu.co; 3 . Universidad CES. Medellín. Colombia. Email: asalazar@ces.edu.co Universidad CES Universidad CES Medellín Colombia asalazar@ces.edu.co

**Keywords:** Estilo de Vida Saludable, Anciano, Modelos Teóricos, Evaluación de Procesos y Resultados en Atención de Salud, Enfermeros de Salud Comunitaria, Healthy life style, Aged, Theoretical Models, Outcome and Process Assessment, Health Care, Nurses, Community Health, Estilo de vida saudável, Idoso, Modelos Teóricos, Avaliagáo de Processos e Resultados em Cuidados de Saúde, Enfermeiros de Saúde Comunitária

## Abstract

**Introducción:**

Este artículo proporciona un aporte importante desde la aplicación del Modelo de Adaptación de Callista Roy a grupos.

**Objetivo:**

Promover comportamientos adaptativos a través de estrategias de atención primaria en salud dirigidas a un grupo de adultos mayores con enfermedad crónica que habitan en un municipio del departamento de Cundinamarca Colombia.

**Materiales y Métodos:**

Abordaje cualitativo tipo investigación-acción participativa. La enfermera utilizó la observación participante, y caracterizó la comunidad; además, con preguntas exploratorias les permitió reconocer sus problemas y proponer estrategias de mejora, incluso para su propia salud; posteriormente se aplicó el proceso de atención de enfermería.

**Resultados:**

Se logró valorar el comportamiento del grupo a través de los modos físico, auto-concepto o identidad grupal, la función del rol o unidad de funcionamiento de la sociedad e integridad social y la interdependencia o contexto social en el que funciona el grupo.

**Discusión:**

Aplicar el Modelo en la práctica permite reconocer situaciones negativas en los grupos para favorecer procesos de afrontamiento innovadores y controlar los estímulos ambientales en el contexto comunitario. Las habilidades en la valoración deben ser agudas ya que son la clave para la aplicación del modelo de Roy en la práctica comunitaria.

**Conclusiones:**

Se identificaron los estímulos que desencadenan los principales problemas de adaptación y se implementó un plan de cuidado con el desarrollo de estrategias para la adaptación, tales como la visita domiciliaria, encuentros intergeneracionales, participación comunitaria con grupos de apoyo creados por la municipalidad y la educación en salud con el fin de promover la adaptación del grupo.

## Abstract (en)

**Introduction:**

This article is an important contribution to the application of Callista Roy's Adaptation Model to groups.

**Objective:**

To promote adaptive behaviors through primary health care strategies for a group of chronically ill older adults living in a municipality in the department of Cundinamarca, Colombia.

**Materials and methods:**

A qualitative participatory action research approach was used. A nurse used participant observation and characterized the community; besides, with exploratory questions, the nurse enabled them to identify their problems and suggest strategies for improvement, including for their own health. Subsequently, the nursing care process was applied.

**Results:**

Group behavior was assessed through the following modes of adaptation: physiologic adaptation, self concept or group identity, role-function adaptation or unit of social functioning and social integrity, and interdependence adaptation or social context in which the group functions.

**Discussion:**

Applying Roy's model in practice makes it possible to identify negative situations in groups, promote innovative coping processes, and control environmental stimuli in a community. Assessment skills must be sharp, as they are key to applying Roy's model to community practice.

**Conclusions:**

The stimuli that trigger the main adaptation problems were identified, and a nursing care plan was implemented developing adaptation strategies, such as home visits, intergenerational meetings, community participation with support groups created by the municipality, and health education, to promote group adaptation.

## Resumo

**Introdujo:**

Este artigo oferece uma contribuido importante da aplicado do Modelo de Adaptado de Callista Roy a grupos.

**Objetivo:**

Promover comportamentos adaptativos por meio de estratégias de atengáo primária a saúde voltadas para um grupo de idosos com doengas crónicas que vivem em um município do departamento de Cundinamarca, Colómbia.

**Materiais e métodos:**

Abordagem de pesquisa de agáo participativa qualitativa. A enfermeira utilizou a observado participante e caracterizou a comunidade; além disso, perguntas exploratórias permitiram que eles reconhecessem seus problemas e propusessem estratégias de melhoria, inclusive para sua própria saúde; posteriormente, foi aplicado o processo de assistencia de enfermagem.

**Resultados:**

Foi possível avaliar o comportamento do grupo por meio de modos físicos, autoconceito ou identidade do grupo, fundo do papel ou unidade de funcionamento da sociedade e integridade social e interdependencia ou contexto social no qual o grupo funciona.

**Discussao:**

A aplicado do Modelo na prática permite o reconhecimento de situagóes negativas em grupos para favorecer processos inovadores de enfrentamento e controlar os estímulos ambientais no contexto da comunidade. As habilidades de avaliagáo devem estar afiadas, pois sáo a chave para a aplicado do Modelo de Roy na prática comunitária.

**Conclusóes:**

Os estímulos que desencadeiam os principais problemas de enfrentamento foram identificados e um plano de cuidados foi implementado com o desenvolvimento de estratégias de enfrentamento, como visitas domiciliares, encontros intergeracionais, participagáo comunitária com grupos de apoio criados pelo município e educagáo em saúde para promover o enfrentamento em grupo.

## Introducción

El aumento de la esperanza de vida, la necesidad de una mejor atención sanitaria y la adopción de comportamientos poco saludables han desencadenado cambios en el patrón epidemiológico con el incremento de las enfermedades crónicas no transmisibles (ECNT )^1^.

Según la Organización Mundial de la Salud (OMS), éstas ocupan las primeras causas de morbi-mortalidad y discapacidad en el mundo, repercutiendo en lo social, económico y sanitario. Aproximadamente el 60,3% de las defunciones en 2017 en el mundo se dieron como consecuencia de padecer una enfermedad crónica, con una incidencia del 50% en adultos mayores^1^ y evidenció consecuencias negativas como dependencia, impotencia, incertidumbre, cambio de roles y disminución en las habilidades físicas y sociales. Por lo tanto, el abordaje a las personas con enfermedades crónicas es un reto, al tener que diseñar acciones que orienten de manera multidimensional la atención sanitaria en la vejez por las implicaciones que tiene para la persona, su familia y la sociedad^2^.

Atendiendo las necesidades de los adultos y adultos mayores con ECNT, en los últimos años se han venido desarrollando acciones para mantener o mejorar el bienestar de esta población^3^; sin embargo, es necesario empoderarlos hacia la cultura del cuidado de sí mismos y la búsqueda de diferentes mecanismos de afrontamiento que les permita adaptarse a un mundo más cambiante y generador de estímulos tanto internos como externos que condicionan el estado de salud. Fomentar comportamientos saludables para prevenir el desarrollo o complicaciones por ECNT, así como la adherencia a sus tratamientos, es prioritario^4^.

El abordaje a las personas mayores implica un trabajo profesional e interdisciplinario; la enfermera/o direcciona el cuidado; para ello durante la última década se han dedicado a implementar en el desarrollo de su práctica, diferentes perspectivas teóricas que representan la esencia del conocimiento, las metas y las actividades de enfermería que incorporan las creencias, valores y conceptos que dan sustento al cuidado del individuo, la familia y la comunidad^5^.

Pocos son los modelos teóricos que orientan el trabajo en grupos; el Modelo de Adaptación de Callista Roy (MAR), permite un acercamiento a los seres humanos tanto individual como colectivamente y los describe como sistemas adaptativos holísticos^6^. Para el presente trabajo se empleó este Modelo, cuya meta es lograr la adaptación a través de la valoración del comportamiento y de estímulos, la identificación de los problemas de adaptación, la implementación de unas metas de cuidado, la ejecución de un plan de cuidado y la evaluación de éste para validar el nivel de adaptación no sólo del individuo sino de los grupos.

El papel de enfermería en salud comunitaria con el abordaje teórico disciplinar, resulta relevante para modular estímulo, generar respuestas efectivas o adaptativas a través del uso de estrategias de atención primaria en salud como educación, motivación a la participación, trabajo intergeneracional, conocimiento de las políticas públicas, e integración de la población a las redes de apoyo social y comunitario^7^^,^^8^.

Las evidencias muestran un uso limitado de abordajes teóricos como el de Roy, en la práctica comunitaria y aún más direccionadas a grupos comunitarios como de adultos mayores con ECNT; A través de la revisión de literatura se identificaron tres artículos que aplican el MAR en grupos; el de Villareal^9^, proporciona una visión general del MAR al cuidar a un grupo de 7 mujeres que estaban dejando de fumar; el estudio de Chen et al^10^ evaluó la salud nutricional de un grupo de adultos mayores y concluyó que se pueden promover conductas adaptativas apoyando los factores biopsicosociales de la población, que influenciaron la situación; y, finalmente Buckner et al^11^ analizaron una comunidad en estado de postcrisis en Arabia, a partir del MAR y la Teoría de adaptación a eventos de la vida, logrando una mayor comprensión de su situación.

Roy argumenta que se debe promover la salud en cada uno de los cuatro modos, manteniendo las respuestas adaptativas y controlando las inefectivas en individuos y grupos para identificar los problemas de adaptación e implementar una atención que la promueva. El proceso de enfermería en el MAR implica: evaluación del comportamiento, de estímulos, formulación del diagnóstico, establecimiento de metas u objetivos, intervenciones y evaluación^12^.

Los grupos son vistos como sistemas, compuestos por partes, e influenciados por variables internas y externas que permiten entender aspectos relevantes que dan cuenta de la salud y estado de una comunidad^13^.

La valoración es fundamental con grupos, no solo los comportamientos y recursos de la comunidad, sino también la adecuación y el funcionamiento de los mecanismos de afrontamiento (estabilizador e innovador)^14^; por tanto, se debe familiarizar con las necesidades de las comunidades utilizando observación, medición, entrevistas para obtener datos de comportamiento que permitan una caracterización de la comunidad e identificar necesidades, posibles riesgos, recursos de salud y apoyo por el equipo interdisciplinario^15^.

Por todo lo anterior el objetivo del presente artículos es presentar la promoción de comportamientos adaptativos a través de estrategias de atención primaria en salud dirigidas a un grupo de adultos mayores con enfermedad crónica que habitan en el municipio de Chía departamento de Cundinamarca Colombia

## Materiales y Métodos

Investigación-acción participativa que permite la reflexión y actuar sobre los problemas derivados de la realidad^16^ y reconocer la situación de las personas mayores con ECNT del municipio de Chía - Cundinamarca - Colombia Los participantes fueron reclutados a través de eventos en salud realizados en la comunidad durante las prácticas formativas de estudiantes de enfermería y fisioterapia, por cuatro meses. Se convocó a los adultos mayores de 55 años o más a participar de sesiones abiertas, con al menos una enfermedad crónica diagnosticada e independientes en su cuidado, sin alteraciones en el estado mental.

La enfermera utilizó la observación participante y caracterizó la comunidad y con preguntas exploratorias les permitió reconocer sus problemas y proponer estrategias de mejora, para su propia salud^17^. La información se analizó de forma manual por las investigadoras y se encuentra almacenada en Mendeley data^18^.

Así se reconocieron las necesidades de los adultos mayores y el compromiso para disminuir comportamientos de riesgo y apropiar conductas de adaptación tanto individuales como grupales. Luego se procedió a identificar los 4 modos adaptativos en relación con los grupos propuestos en el MAR.

Finalmente, durante el segundo semestre de 2018, se aplicó el proceso de enfermería a través de la visita domiciliaria periódica (cada mes durante 8 meses consecutivos), actividades intergeneracionales de participación comunitaria, educación en salud, motivación a la participación en sesiones programadas por la municipalidad para promover la adaptación al grupo, en orden a reforzar los comportamientos de riesgo identificados por ellos durante las sesiones. Se aplicaron las consideraciones éticas planteadas en la Resolución 843019 de 1993, se solicitó el consentimiento informado, el aval institucional, permisos con la municipalidad y para la toma de registros fotográficos, se respetaron los principios éticos de autonomía, justicia y se contempló la política de protección del medio ambiente.

## Resultados

### Caracterización de la población

Participantes: 150 adultos mayores con ECNT residentes en Chía. El 60 %, mujeres el 52% casados, el 27% viudos y el 21 % entre separados y solteros el 72% tenía vivienda propia, el 28 % alquilada en relación con el nivel educativo la mayoría tenía primaria incompleta y completa. Los principales diagnósticos de Enfermedad crónica fueron hipertensión (HTA), 79%, artritis degenerativa 3% y diabetes mellitus tipo 2 (DM2), 18%; los años con el diagnóstico, oscilaban entre 1 y 3 años y siete y veinte años.

### Proceso de enfermería en la comunidad de adultos mayores con ECNT^20^

Se valora comportamientos, recursos, adecuación y mecanismos de adaptación (estabilizador e innovador); como las necesidades de la comunidad en la cual está aplicando el modelo^14^. Se identifican los comportamientos inefectivos en los modos de adaptación como las necesidades básicas de la comunidad a partir de los indicadores generales de los grupos en relación con los sistemas comunitarios y las necesidades en ambientes cambiantes donde se producen respuesta frente a satisfacer las necesidades de un grupo^13^.

Modo físico: Se define como la manera en que el sistema adaptativo humano manifiesta adaptación con recursos operativos básicos, que incluyen los miembros de la comunidad, las familias, las organizaciones la sociedad en general tanto a nivel local como global.

Roy identifica las capacidades de los miembros del grupo como un recurso importante para los sistemas adaptativos humanos colectivos, donde se espera que participen activamente, así como identificación de los recursos físicos, que incluyen el conocimiento, las habilidades, los compromisos, las relaciones y la salud de los individuos dentro del colectivo^6^, también el reconocimiento de los recursos fiscales.

Las áreas de evaluación en el modo físico incluyen, los miembros de la comunidad y sus capacidades de adaptación, así como las preocupaciones expresadas, que permiten identificar prácticas personales para la prevención de enfermedades y la promoción de la salud.

Para identificar las necesidades del grupo se llevaron a cabo cuatro encuentros con los adultos mayores que evidenciaron cómo desestiman la percepción de su estado de salud, desconociendo las complicaciones y riesgos asociados a su enfermedad, expresan olvidar tomar los medicamentos en los horarios establecidos, el no realizar actividad física por falta de tiempo o carencia de espacios cercanos y suficientes para realizarlos, así como el no contar con los recursos económicos para adquirir los alimentos que son formulados por el médico.

**Figura 1:**
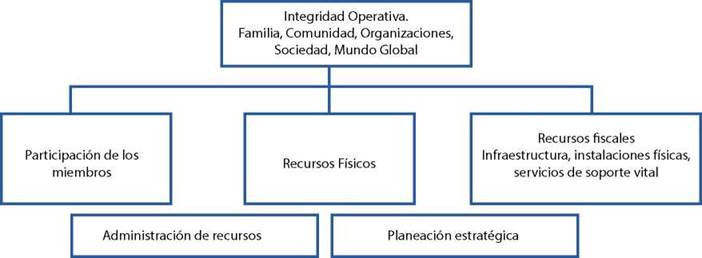
Modo Adaptativo Físico en Grupos

Fuente: Tomado de Roy C,1999^6^

Refieren no contar con suficiente ayuda de los familiares cercanos, lo que lleva a generar en ellos sentimientos de soledad, además no perciben seguimiento por parte del sistema de salud.

A través de las visitas domiciliarias se observó las características de las viviendas, algunos poseen viviendas propias, otros, arrendadas y algunos viven con sus familiares. En relación con las condiciones físicas cuentan con servicios básicos, agua, luz, iluminación ventilación y estados de paredes, pisos y techos aceptables, el promedio de habitante por vivienda es de 5 personas para quienes viven acompañados, y otros solos. Se resalta la carencia de hábitos higiénicos de las viviendas, la presencia y convivencia con animales domésticos (perros, gatos, gallinas, pájaros), mal manejo de residuos y presencia de zoonosis. Recursos físicos de baja capacidad para la atención y seguimiento del adulto mayor por parte del sistema de salud. Falta de espacios para recreación y socialización.

Identidad grupal: Es un resultado de los modos adaptativos grupales, se construye a partir de la autoimagen grupal compartida; que se logra a partir de las relaciones interpersonales de los miembros del grupo, se relaciona con el contexto, valores, creencias, aspectos comunes como economía, la política, la religión, la familia que hacen parte de la cultura y el contexto social donde interactúan.

**Figura 2:**
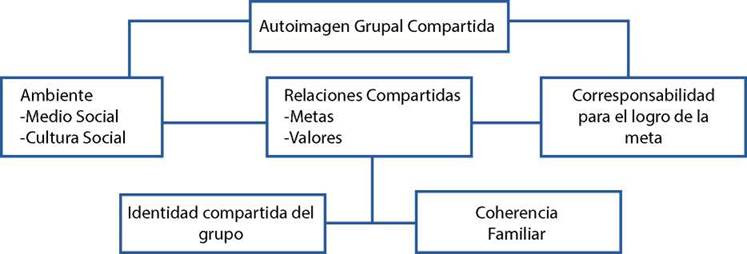
Modo de integridad grupal

Estas relaciones permiten forjar normas y metas comunes en beneficio del grupo y genera una corresponsabilidad con el desarrollo de éstas. Lo anterior hace parte del modo de identidad de grupo (ver Figura 2), que para su evaluación incluyen, la evaluación de la identificación cultural de los miembros de la comunidad, la solidaridad entre los miembros; así como la presencia o ausencia de grupos comunitarios previamente organizados cuyas metas reflejan mejoras para la comunidad^12^.

Fuente: Tomado de Roy C,1999^6^

A través de las sesiones grupales los mayores manifiestan, como sus relaciones con amigos han disminuido por factores como cambio de residencia, dependencia de sus hijos por su enfermedad crónica, fallecimiento de compañeros, situaciones de salud que les impide acudir a actividades sociales, por cambios propios del envejecimiento como disminución de la agudeza visual y auditiva, dificultades en la movilidad por uso de elementos ortésicos, perciben que la comunidad los ven como una carga y sienten que ya no son útiles y vulnerables para la sociedad.

**Figura 3:**
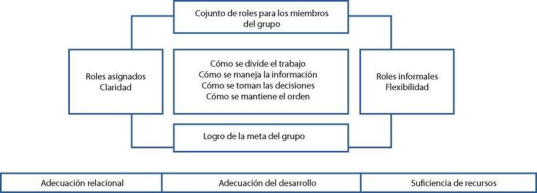
Modo de Rol

Función del Rol: se relaciona con la necesidad de comprender y comprometerse a cumplir una tarea específica dentro de un grupo. Cada miembro tiene un rol específico que le permite cumplir unas funciones para alcanzar los objetivos comunes, y la misión propuesta^6^. Se evalúa la eficacia de las instituciones que protegen y sirven a la comunidad, como el departamento de policía, de bomberos, los hospitales entre otros. (ver Figura 3)

Además, tiene que ver con los roles que el individuo tiene en la sociedad, y el conjunto de expectativas sobre cómo se ocupa una posición y se comporta. Engloba siete subdimensiones: A. rol primario: edad, sexo y estado de desarrollo. Determinan los comportamientos en un determinado período de crecimiento de la persona. B. Rol secundario: cuando asume tareas asociadas con el estado del desarrollo mental y rol primario. C. Rol terciario: seleccionado libremente por la persona, es temporal y se asocia a tareas menores en el desarrollo de la persona. D. Comportamiento instrumental: o con objetivo. Actividades del rol que la persona ejecuta. E. Comportamiento expresivo: sentimientos y actitudes que ayudan a la persona a la ejecución del rol. F. Tomando el rol: es una interacción o un juicio sobre otro rol. Se enfoca en el significado que los actos tienen para las personas en la interacción del rol. G. Integrando roles: son los diferentes roles y sus expectativas.

Fuente: Tomado de Roy C, 1999^6^

Se identificó que los adultos del grupo tienen como roles principales ser abuelos, padres, suegros, amigos y especialmente cuidadores de nietos y cónyuges; sin embargo, en cuanto a la integridad social ellos manifiestan tener la oportunidad de pertenecer a grupos representativos de adultos mayores aunque su participación sea limitada; una de las actividades donde tienen la oportunidad de participar es el comité gerontológico municipal, pero solo pueden estar aquellas personas que poseen grandes cualidades de liderazgo.

Así mismo, hay participación en grupos de apoyo derivados de su condición (club de hipertensos, diabéticos, etc.) donde su presencia es corta, por ser programadas durante 30 minutos con un intervalo mensual. Se hace necesario resaltar que, aunque existe un hospital local que ofrece acciones en salud a grupo de adultos mayores, no son suficientes debido a la baja cobertura.

Modo Interdependencia: se relaciona con tres componentes: el contexto en el que opera, la infraestructura y las personas que participan. El contexto se refiere a influencias externas (económicas, políticas, culturales) e internas (misión, visión, valores). La infraestructura se refiere a los procesos afectivos, de recursos y de desarrollo que existen dentro de las relaciones de los individuos entre sí. Las personas constituyen el tercer componente del modo de interdependencia, donde se evidencian las habilidades de afrontamiento, como actitudes, habilidades y compromisos.

**Figura 4:**
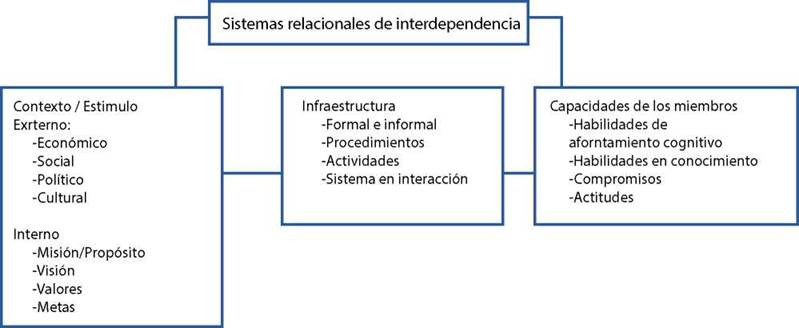
Modo de Interdependencia

Las áreas de evaluación incluyen la colaboración entre la comunidad, las organizaciones cercanas y profesionales externos, que apoyan a los miembros de la comunidad. Este llevaría a alcanzar la adecuación relacional, de desarrollo y recursos, características primordiales en este modo^14^. (ver Figura 4)

Fuente: Tomado de Tomado de Roy C,1999^6^

El familiograma y el ecomapa permitieron identificar la infraestructura, el contexto social y los recursos con los que cuentan los individuos dentro del grupo. Se evidenció que, aunque hay un gran porcentaje de adultos mayores que viven solos, y no requieren cuidador, refieren buscar soporte en vecinos, juntas de acción comunal, grupos de apoyo organizados por las autoridades municipales, donde se crean vínculos de amistad, de acuerdo con sus afinidades personales.

Otro aspecto relacionado con el contexto y los recursos hace referencia a la percepción de relaciones que se fomentan a través de los programas coordinados por la alcaldía municipal, quien delega a un secretario de desarrollo social para propiciar actividades de ocupación del tiempo libre que favorecen la integración y socialización.

*Identificación de respuestas.* Se identificaron varios comportamientos en el grupo como el incumplimiento en la toma de medicamentos, consumo de alimentos no saludables, sedentarismo, sobrepeso, incumplimiento de citas médicas, poca comunicación con el personal de salud, pasividad durante las consultas, percepción de soledad en cuanto al cuidado de la salud, sentimientos de minusvalía, autocompasión, percepción de baja autoestima, poca participación, falta de liderazgo, victimización, percepción de poco apoyo de las autoridades locales, desconfianza institucional, problemas fisiológicos comunes como disminución de la visión, audición y capacidad motriz, disminución de la memoria reciente, entre otros.

*Valoración del comportamiento* permitió identificar los recursos básicos y físicos del grupo de adultos mayores, en su mayoría de estratos socioeconómicos bajos, viven en áreas rurales, aún dependen de trabajos informales, pertenecen al régimen subsidiado del sistema general de seguridad social, participan en las actividades que ofrece la municipalidad para el beneficio de los adultos mayores; en cuanto a la identidad grupal, perciben que mantienen buena relación con las autoridades locales y entre sí; sin embargo, se consideran una carga social que requieren de mayor apoyo por parte de sus familias y de las políticas de gobierno; culturalmente, sienten que son vistos como personas poco productivas; como unidad de funcionamiento de la sociedad, están organizados y participan del comité gerontológico municipal, y en cuanto al contexto social en que funciona el grupo cuentan con el centro día donde se pueden reunir y compartir experiencias, habilidades y expresar sentimientos y la ocupación del tiempo libre.

*Identificación de los estímulos*. se determinan los estímulos que causan comportamientos (focales, contextuales, residuales) y que influyeron en la identificación del problema. La identificación del nivel de adaptación de la comunidad y en particular, cualquier proceso de vida compensado o comprometido manifestado por el comportamiento de la comunidad, es parte de la evaluación de estímulos. La identificación de estímulos que afectan el comportamiento de una comunidad puede ser bastante complejo de identificar debido a las múltiples influencias ambientales que experimentan las comunidades y sus miembros^14^.

El estímulo focal identificado en este grupo de adultos mayores con enfermedad crónica que han influenciado los comportamientos en los cuatro modos identificados anteriormente es la baja percepción que tiene el grupo acerca de la importancia de su estado de salud lo que conlleva a un desconocimiento de complicaciones propias de su enfermedad de base, evidenciado en el no cumplimiento en sus tratamientos y con la falta de estrategias de afrontamiento para adaptarse a su enfermedad crónica.

Estímulos contextuales: poco soporte familiar evidenciado por falta de acompañamiento de familiares o cuidadores en la asistencia a los sistemas de salud, escasas redes de apoyo social y del sistema de salud, soledad, sistema de protección ineficiente, con baja cobertura para el cubrimiento de necesidades básicas (inestabilidad económica, alimentación, vivienda), falta de programas ofrecidos por el municipio, por el hospital local y por instituciones privadas para apoyo a su condición de salud. Falta de motivación por mantener buenos hábitos higiénicos de los entornos donde habitan. Pobres canales de comunicación de las autoridades locales. Poca voluntad política para los programas de adulto mayor.

*Diagnóstico de enfermería* refleja la relación entre los comportamientos y los estímulos. Es un proceso de juicio que resulta en declaraciones que transmiten el estado de adaptación del sistema humano^6^. Los comportamientos observados y los estímulos influyentes más relevantes constituyen un diagnóstico de enfermería.

Mantenimiento ineficaz de la salud del grupo relacionada con déficit de conductas de adhesión a su condición evidenciado con baja percepción de salud, dificultad en el acceso a los servicios de salud, escasas redes de apoyo social, bajo soporte familiar, desmotivación, soledad, baja participación en actividades sociales e incumplimiento de sus tratamientos.Metas. Implica tener claridad sobre los resultados que se quieren alcanzar para promover la adaptación. La forma en que cambiarán los comportamientos y el marco de tiempo en el que se debe alcanzar, en las comunidades pueden requerir meses o incluso años para lograrlo^13^.

La meta a mediano plazo. Desarrollar estrategias de afrontamiento en el grupo de adultos mayores con enfermedad crónica que permitan adoptar conductas de adhesión a su condición de salud.

Intervenciones. Hay una necesidad de brindar apoyo a la vida personal y familiar. Las intervenciones se enfocaron en apoyar la adaptación positiva y reforzar estrategias integradas de afrontamiento a los miembros de la comunidad; que, a través de las sesiones de apoyo, reconocieron medidas de afrontamiento ineficaces y cambiar estrategias para una estabilización y recuperación efectiva^21^.

Las intervenciones comunitarias pueden dirigirse a mejorar los mecanismos de afrontamiento y/o a manejar estímulos^12^. Las intervenciones realizadas al grupo fueron:

1. Visitas domiciliarias interdisciplinarias a los 150 adultos mayores, cada mes durante 8 meses, dando educación en salud frente a las necesidades reportadas por ellos durante las sesiones grupales y ahora direccionadas a su propio contexto, los temas para educación en salud fueron hábitos nutricionales, actividad física y cumplimiento de tratamiento farmacológico, ajustado a los propios requerimientos y recursos, con el fin de lograr despejar dudas, generar conocimientos y desarrollar autonomía para el cuidado de su salud.
2. Se realizaron actividades intergeneracionales cada tres meses donde el grupo de adultos mayores ECNT y un grupo de estudiantes de los programas de enfermería y fisioterapia se reunían para trabajar aspectos relacionados con roles sociales, experiencias de vida que generan aprendizajes en doble vía. Los adultos aprenden de los jóvenes su cultura, su nueva forma de pensar y los jóvenes aprenden a partir de las experiencias de los adultos a mejorar aspectos relacionados con su vida que trascienden al contexto personal y familiar.
3. Trabajo en cooperación con otros grupos de apoyo. Estas actividades se desarrollaron en conjunto con grupos del municipio como por ejemplo recreación y deporte, la secretaría de cultura y bienestar, las cuales dieron soporte a los grupos vinculados a actividades que favorecen su bienestar y potencializaron sus capacidades a partir de actividades ocupacionales.
4. Actividades de participación comunitaria apoyados en los programas radiales de la comunidad, donde ellos pueden compartir sus experiencias de salud, para motivar e incentivar a otros que estén viviendo experiencias similares, para que puedan tener comportamientos de cambio. Así mismo, aprovechan este medio para vincular al grupo a actividades organizadas por los líderes del mismo grupo por ejemplo paseos, rifas, bazares, etc.
5. Educación en salud, para esto el grupo de enfermeras y fisioterapias construyeron ayudas didácticas como folletos, videos, cartillas, de acuerdo con las necesidades sentidas de la población con el fin de orientar comportamientos saludables y mantenerlo en el tiempo cuando no podían estar con ellos.

A medida que las personas desarrollan relaciones y nuevas interacciones de apoyo y empatía, se fueron empoderando mientras trabajaban con el equipo de práctica y con sus compañeros, lo que fue dando resultados positivos^21^. Estas estrategias son parte del subsistema estabilizador. La identidad comunitaria también tomó forma a través del subsistema y las intervenciones realizadas aumentó la motivación. Así los recursos, valores compartidos, claridad de roles e integridad relacional crecieron, demostrando crecimiento en los cuatro modos adaptativos^21^.

Evaluación. Allí se determina si las intervenciones y los esfuerzos para mejorar los estímulos fueron efectivos para alcanzar las metas establecidas. Se deben interpretar los hallazgos encontrados antes y después de los encuentros grupales, visitas domiciliarias encuentros intergeneracionales y grupos de apoyo, entre otros, para valorar si existieron modificaciones en sus comportamientos a partir de las estrategias desarrolladas^14^.

Al finalizar las visitas domiciliarias programadas durante los ocho meses se realizó una entrevista a los adultos mayores, y se preguntó cómo se habían sentido con esta estrategia, ellos refirieron sentirse motivados al tener el acompañamiento mensual por parte del equipo, despejaron dudas, generó seguridad y cambios positivos en su salud al bajar de peso, regular su tensión arterial y lograr adquirir una rutina de actividad física que, además, favoreció la socialización y los hizo crear conciencia de la necesidad de generar conductas de autocuidado.

La primera caracterización de la comunidad y durante las visitas domiciliarias se aplicó una lista de chequeo la cual permitió reportar lo observado en el contexto externo antes y después del desarrollo de las visitas domiciliarias, para evidenciar si existieron mejoras en el entorno donde viven, características de la vivienda en relación al estado de paredes, techos, iluminación, ventilación servicios públicos e higiene y, efectivamente, se logró identificar una mejora en estos recursos dados por un mejor saneamiento, y aseo de la vivienda, disminuyendo factores riesgo para ellos como para la comunidad .

Durante el cierre de las actividades de participación comunitaria el grupo manifestó haber aprendido de las experiencias de otros. También sienten que pudieron dar mucho de sí mismos especialmente a los jóvenes en aspectos relacionados con la cultura y los valores.

Manifestaron satisfacción en doble vía: adultos mayores con enfermedad crónica y jóvenes al compartir experiencias relacionadas con la salud y la enfermedad, ya que esta estrategia generó conciencia en los jóvenes para el autocuidado y los comportamientos saludables.

## Discusión

Roy ha identificado dos procesos de afrontamiento para el ser humano: sistema social que incluye el subsistema estabilizador y el subsistema innovador. Ambos dirigidos al mantenimiento del sistema e involucra la estructura establecida, los valores y actividades diarias mediante las cuales los participantes logran contribuir a los propósitos comunes de la sociedad^6^. Ayuda a prevenir el desequilibrio cuando se está introduciendo en una familia, una comunidad o un grupo de trabajo. El subsistema innovador ofrece estructuras y procesos para el crecimiento y cambio^6^ y tiene un contorno dinámico e involucra procesos que dan lugar a relaciones familiares, comunitarias u otros sistemas sociales. Por tanto, a través de las actividades realizadas como la consejería familiar, planificación estratégica, trabajo en equipo, u otros tipos de actividades grupales diseñadas, facilitaron un mayor nivel de funcionamiento relacional^22^.

Aplicaciones del modelo en la práctica: los subsistemas estabilizador e innovador fueron importantes para este ejercicio práctico ya que se vieron beneficios al ayudar a los adultos mayores cuando estaban con situaciones negativas de estímulos ambientales en la unidad, además los concientizó de su potencial a través de procesos de afrontamiento innovadores^22^.

Aunque la literatura revela un uso limitado del modelo en la comunidad, es adecuado para la salud comunitaria, ya que explica las relaciones entre los participantes y sus entornos, las áreas para la evaluación de la enfermería de salud comunitaria y el papel de la enfermera en la prestación de atención a grupos de personas. Aunque el modelo de Roy se ha categorizado como complejo y se ha cuestionado su idoneidad para el proceso de evaluación de la comunidad, se ha encontrado que el modelo es capaz de describir y predecir la naturaleza compleja de los procesos ineficaces, interacciones persona - entorno.

Una de las verdaderas fortalezas se relaciona con su naturaleza teórica ya que ofrece orientación conceptual para avanzar en la práctica y el conocimiento de la enfermería de salud comunitaria sin una estructura rígida para la recopilación de datos en la que se basan muchos otros modelos de práctica de enfermería de salud comunitaria y herramientas de evaluación comunitaria. Las habilidades en la valoración deben ser muy agudas, ya que son la clave para la aplicación del modelo de Roy a la práctica de enfermería en la salud comunitaria^14^.

Otros estudios como el de Herrera Santí et al, muestra los resultados de una estrategia de intervención para mejorar los niveles de calidad de vida, en adultos mayores. El proceso conto con tres momentos: el primero valoro a través de la escala MGH elaborada y validada en Cuba la calidad de vida. Luego realizaron una intervención educativa que incluida ejercicios de preparación física, relajación, dinámicas grupales para modificar actitudes, bailo terapia, técnica de buen consejo, charlas educativas, auto masajes a través de 12 sesiones de 2 horas de duración. En el tercer momento se realizó reevaluación con el mismo instrumento. Después de la intervención se pudo observar una variación importante en los niveles de autoestima, igualmente favoreció los estilos saludables, aumento el número de personas con alta calidad de vida^23^.

En cambio, el trabajo de grado de Serrano Romero tuvo como objetivo apoyar la intervención psicosocial de los adultos mayores y funcionarios del Centro de Bienestar del Anciano, Juan Pablo II de Floridablanca (CBA JPII), utilizando los instrumentos Mini-Examen del Estado Mental (MMSE), el Cuestionario breve del dolor (CBD) y formatos de evaluación cuantitativos y cualitativos. Encontrando resultados heterogéneos en el CBD, el resultado promedio del MMSE en la evaluación inicial fue de 17/30 puntos y en la segunda evaluación correspondió a 18,86/30 puntos y las calificaciones de los formatos de evaluación en su mayoría corresponden a excelente^24^.

Lee LL et al., tuvo como objetivo examinar las formas en que la teoría de la autoeficacia podría usarse en programas de intervención diseñados para superar las barreras psicológicas para aumentar la actividad física entre las personas mayores, concluyeron que la evidencia de algunos ensayos respalda la opinión de que es beneficioso incorporar la teoría de la autoeficacia en el diseño de una intervención de actividad física. Las intervenciones de actividad física dirigidas a mejorar la autopercepción de la autoeficacia del ejercicio pueden tener efectos positivos sobre la confianza y la capacidad para iniciar y mantener el comportamiento de actividad física. Las enfermeras tienen varias formas de ayudar a las personas mayores a aprovechar las cuatro fuentes de información de la autoeficacia: logros de desempeño, aprendizaje indirecto, estímulo verbal y estados fisiológicos y afectivos^25^.

Refieren Prajankett O et al., preparar enfermeras para trabajar en la comunidad es un requisito previo para satisfacer las necesidades de salud de la población que envejece de manera sostenible. Las oportunidades de educación, desarrollo profesional y capacitación en liderazgo deben centrarse en el desarrollo de capacidades para: a) fortalecer la responsabilidad mutua, b) reorientar el entorno laboral a través de modelos de atención innovadores y c) coordinar servicios a través de alianzas para lograr la salud universal y garantizar un envejecimiento saludable^26^.

Así mismo la promoción de comportamientos efectivos de autocuidado y autocontrol son fundamentales para mejorar los resultados de las enfermedades crónicas. Es probable que la adaptación y focalización de las intervenciones apropiadas para las personas y las comunidades sea más eficaz para impulsar el cambio de comportamiento. Esta revisión ha identificado que el establecimiento mutuo de objetivos mejoró los comportamientos de salud. La flexibilidad para adoptar intervenciones de autocuidado en entornos comunitarios mostró mejores resultados para los pacientes^27^.

## Conclusiones

La aplicación de un marco teórico en la práctica, como MAR en grupos, permite avanzar en el conocimiento de la salud comunitaria beneficiando diversos grupos poblacionales, así como guiar el trabajo de estudiantes, profesionales e investigadores en la generación de conocimiento para aplicar intervenciones.

La promoción de la salud y la prevención de la enfermedad, han estado presentes a lo largo de la historia, y han involucrado tanto al individuo como a la comunidad, de esta manera la salud pública comunitaria tiene un enfoque más proactivo y preventivo de acercamiento hacia la salud. Desafortunadamente estas prácticas se realizan de forma desarticulada, y no bajo estructuras teóricas que brindan un marco al trabajo comunitario, donde se aumenten los esfuerzos de colaboración interdisciplinaria y la participación comunitaria.

La aplicación del MAR al grupo de adultos mayores con ECNT apoyó el desarrollo de estrategias de afrontamiento indispensables para lograr su adaptación a la situación de cronicidad.

Las personas con ECNT, especialmente la población adulta mayor, encuentra más dificultades para acceder a los recursos ofrecidos por el sistema de salud, ya sea por sus comorbilidades, pluripatologías e incluso por su baja independencia. En ese orden de ideas la demanda de una atención desde el ámbito comunitario se convierte en una necesidad a fin de lograr su empoderamiento.
